# Control of invasive alien plants on an oceanic island modifies invertebrate folivory to the benefit of native biodiversity

**DOI:** 10.7717/peerj.21908

**Published:** 2026-11-12

**Authors:** François M. M. P. Baguette, Ghanishta Seeburrun, Cláudia Baider, François Benjamin Vincent Florens

**Affiliations:** 1 Tropical Island Biodiversity, Ecology and Conservation Pole of Research, Department of Biosciences and Ocean Studies, Faculty of Science, University of Mauritius, Le Réduit, Mauritius; 2 The Mauritius Herbarium, Agricultural Services, Ministry of Agro-Industry, Food Security, Blue Economy and Fisheries, Le Réduit, Mauritius

**Keywords:** Ecological interaction, Ecological restoration, Ecological succession, *Harungana madagascariensis*, Invasive alien plants, Invertebrate herbivores, Mauritius, Plant-animal interaction, *Psidium cattleyanum*, Weed control

## Abstract

Herbivory is one of the most important ecological interactions globally. Herbivores, among which arthropods predominate, play key roles in fundamental ecological processes, and may profoundly affect plants. Invasive alien plants (IAP) may influence herbivory in varied ways, including by supplying additional resources, yet, few comparative studies have assessed how IAP impact herbivory, particularly on heavily invaded oceanic islands. Here, we assess how IAP influences folivory, the prominent form of herbivory, to explore the ecological and conservation implications in a habitat restoration context. We studied a protected native forest on the oceanic island of Mauritius, comprising an area invaded by IAP and an adjacent area weeded of IAP used for comparison. We first quantified IAP impacts on folivory by comparing arthropod communities between invaded and weeded areas using yellow sticky traps set along 100 m transects. We then compared the incidence and severity of folivory (including chewing, galling, and mining) across habitat types on four shared native species between habitats, along with *Psidium cattleyanum*, the dominant IAP in invaded forest, and *Harungana madagascariensis*, the dominant native pioneer tree naturally regenerating in weeded forest. Finally, we compared the specific leaf area (SLA), folivory rate, and consumed leaf area on the selected species between habitats. Arthropod species richness and abundance were comparable between habitats but community structure differed. Overall, herbivory incidence on natives was higher in invaded than weeded area, with *Antirhea borbonica* and *Labourdonnaisia calophylloides* showing higher odds of folivory in invaded forest. Herbivory severity was also higher in the invaded area, with *A. borbonica* showing higher herbivory severity damage, although the opposite was found for *Aphloia theiformis*. There was no significant difference of folivory rate between habitat type, except for *H. madagascariensis* that sustained a significantly higher rate than *P. cattleyanum*. Overall, mean consumed leaf area was higher on *H. madagascariensis* in the weeded area, and *A. borbonica* in the invaded one. Finally, SLA was significantly higher on *H. madagascariensis* compared with *P. cattleyanum*, like on *L. calophylloides* in weeded area. Our study shows that IAP control represents limited loss for folivores. By allowing native pioneer regeneration, IAP control indirectly increases leaf resource availability, and decreases the risk of folivory damage on native species. Conservation strategies should therefore focus more strongly on *in situ* control of IAP, and keep native pioneer species, instead of removing them, as often done on the island. This would strengthen plant-herbivore interactions and enhance benefits for biodiversity. Ultimately, not cutting back pioneer native trees would free conservation resources, allowing the restoration of larger areas, and further improve the effectiveness of conservation measures.

## Introduction

Herbivory, defined as the consumption of living plant tissues by animals, is one of the most important ecological interactions globally (Johnson, 2011; Meyer, Koch & Weisser, 2015). It includes many different forms depending on which plant tissue is being consumed, the most common ones being frugivory (fruit consumption), florivory (flower and flower bud consumption), and folivory (leaf consumption), the latter featuring prominently and being the focus of this study. Herbivores are estimated to consume over 15% of plant biomass produced annually in temperate and tropical ecosystems (Hartley & Jones, 1996; Gong & Zhang, 2014), and in doing so play a key role in fundamental ecological processes such as nutrient cycling and facilitating energy transfer to higher trophic levels (Loreau, 1995; Coley & Barone, 1996; Agrawal, 2011; Schowalter, 2022), thus supporting much of the biodiversity of forests. However, aboveground herbivory can also have negative effects on plants by reducing growth, development, and reproductive output through the disruption of photosynthesis and the loss of nutrients, which may ultimately alter plant fitness, community structure and composition (Frost & Hunter, 2008; Allan & Crawley, 2011), and contribute to shape ecosystem successional trajectories (McBrien, Harmsen & Crowder, 1983; Brown & Gange, 1992; Carson & Root, 1999). Such negative effects on plant fitness, however, also feeds back into fostering species coexistence thereby maintaining high natural diversity (Nascimento & Proctor, 2001; Marquis, 2005) which is of particular importance for biodiversity conservation.

Invertebrates constitute a major group of herbivores, being the most speciose group of animals globally, with approximately half of them feeding on plants (Schoonhoven, Van Loon & Dicke, 2005). They constitute the primary folivores, being responsible for the majority of consumed leaves in forests (Coley & Barone, 1996) and accounting for up to 98% of total leaf loss in tropical forests (Janzen, 1981; Adams et al., 2009; Weisser & Siemann, 2013; Suzuki et al., 2013; Schowalter, 2022). As a result, invertebrates play key functional roles in terrestrial ecosystems like regulating plant performance (Maron, 1998; Delaney & Macedo, 2000), shaping plant community dynamics (Crawley, 2019), and being central in extensive food webs (Novotny et al., 2012; Novotny & Miller, 2014). Despite their importance, these folivores remain far less studied than vertebrate herbivores or pollinators, particularly in tropical systems (Titley, Snaddon & Turner, 2017; Lebbink et al., 2024). This knowledge gap limits our understanding of plant–herbivore interactions and ecosystem functioning, especially in understudied regions like oceanic islands that harbour disproportionately high levels of endemic biodiversity (Motala et al., 2007).

Invasive alien plants (IAP) have been reported to contribute predominantly negatively on herbivory by disrupting established plant–herbivore networks (Harvey, Bukovinszky & van der Putten, 2010), altering herbivore behavior (Awmack & Leather, 2002; Harvey & Fortuna, 2012; Forister & Scholl, 2012), contributing to invertebrate herbivores decline (Tallamy, Narango & Mitchell, 2021), and changing community dynamics (Cronin, 2007; Hein & Gillooly, 2011). However, other studies show neutral or even positive outcomes (Schlaepfer, Sax & Olden, 2011; Maoela et al., 2019), indicating that the indirect effects of IAP on herbivory can be context-dependent, and no consistent global response pattern has yet emerged for the impact of anthropogenic forest structural change on foliar herbivory (Walker & Jones, 2001; Rossetti et al., 2017). Consequently, the impacts of IAP on plant–herbivore interactions require further investigations that are particularly important to address on oceanic islands, which are highly vulnerable to IAP impacts and characterised by simplified interaction networks. On these islands, even slight disruptions may have cascading consequences for plant–herbivore interactions and direct implications for biodiversity conservation (Gillespie, Claridge & Roderick, 2008; Schoener et al., 2010; Zeng et al., 2019).

Mauritius, a small tropical oceanic island, exemplifies this vulnerability as a large proportion of its remaining 4.4% original native forest cover (Hammond et al., 2015) is heavily invaded by IAP (Florens et al., 2016). Previous studies on IAP conducted on the island focused primarily on the effects they have on native plant species (Baider & Florens, 2006; Monty, Florens & Baider, 2013) and plant community composition (Baider & Florens, 2011; Florens et al., 2012, 2017), as well as impacts on native invertebrate communities (Florens, Daby & Jones, 1998; Florens et al., 2010). However, the indirect effects of IAP on ecological interactions, such as folivory, have rarely been investigated (Seeburrun et al., 2026), reflecting a broader paucity of studies on folivory in oceanic island systems (Moreira et al., 2021; Moreira & Abdala-Roberts, 2022). This study investigates how IAP, and their control, impact folivory to identify implications for biodiversity conservation. Our objectives were to: (1) compare arthropod community composition between forest invaded by IAP (hereafter invaded forest) and forest undergoing ecological restoration through removal of IAP (hereafter weeded forest); (2) compare the incidence (proportion of leaves damaged) and severity (%) of folivory on native tree species between the two forest types; and (3) assess the potential effects of IAP on resource availability for folivores by comparing specific leaf area (SLA) and consumed area of selected native species between invaded and weeded forests. The findings of this study will provide evidence of relevance to regional conservation management, which currently often suffer from a persistent deficit of evidence-based management (Florens & Baider, 2013; Baguette et al., 2022; Baguette, Baider & Florens, 2025a).

## Materials and Methods

### Data collection

We did a comparative field study in Plaine Champagne (centered on 20°25′15′S and 57°26′50′E, 700 m asl), within the Black River Gorges National Park (BRGNP) on the volcanic oceanic island of Mauritius (Mascarenes) (Fig. 1) with approval from the Ministry of Agro-Industry and Food Security. It is the only place where we found no evidence of the native pioneer *Harungana madagascariensis* Lam. ex. Poir. (Hypericaceae) being cut down by conservation practitioners (Florens & Baider, 2013; Baguette, Baider & Florens, 2025a, 2025b). It is characterised by a super-humid mesothermal climate receiving an average of 4,000 mm of rain annually (Halais & Davy, 1969), and lateritic soil limiting the native vegetation growth to about 5 m in height (Parish & Feillafe, 1965). The area is highly invaded by Strawberry guava (*Psidium cattleyanum* Sabine (Myrtaceae)), the dominant species in the area (Dulloo et al., 1999), except in adjacent blocks where ecological restoration through IAP removal has been conducted since 2017. We therefore set our sampling design in adjacent blocks where one area was invaded and the other had woody IAP removed (Fig. 1). Habitat conditions in both areas were similar, with variations in microclimate and canopy openness being mostly due to different vegetation structure after the weeding, with soil conditions and site history being the same. At each forest type, arthropods were sampled using ten double-sided yellow sticky traps (22 × 28 cm), placed at 10 m intervals along randomly established transects and left in the field for 1 week. Arthropods were sampled twice, in August 2025 (winter) and January 2026 (summer), to account for seasonal variation. All traps were collected and stored at 6–8 °C until processing. Arthropods captured were counted and examined with the help of a 10× stand magnifier and photographed prior to identification. Identification was done to the Order level and consequently, herbivorous arthropods were retained for later analysis as a surrogate for exclusively folivorous taxa (Material S1).

**Figure 1:**
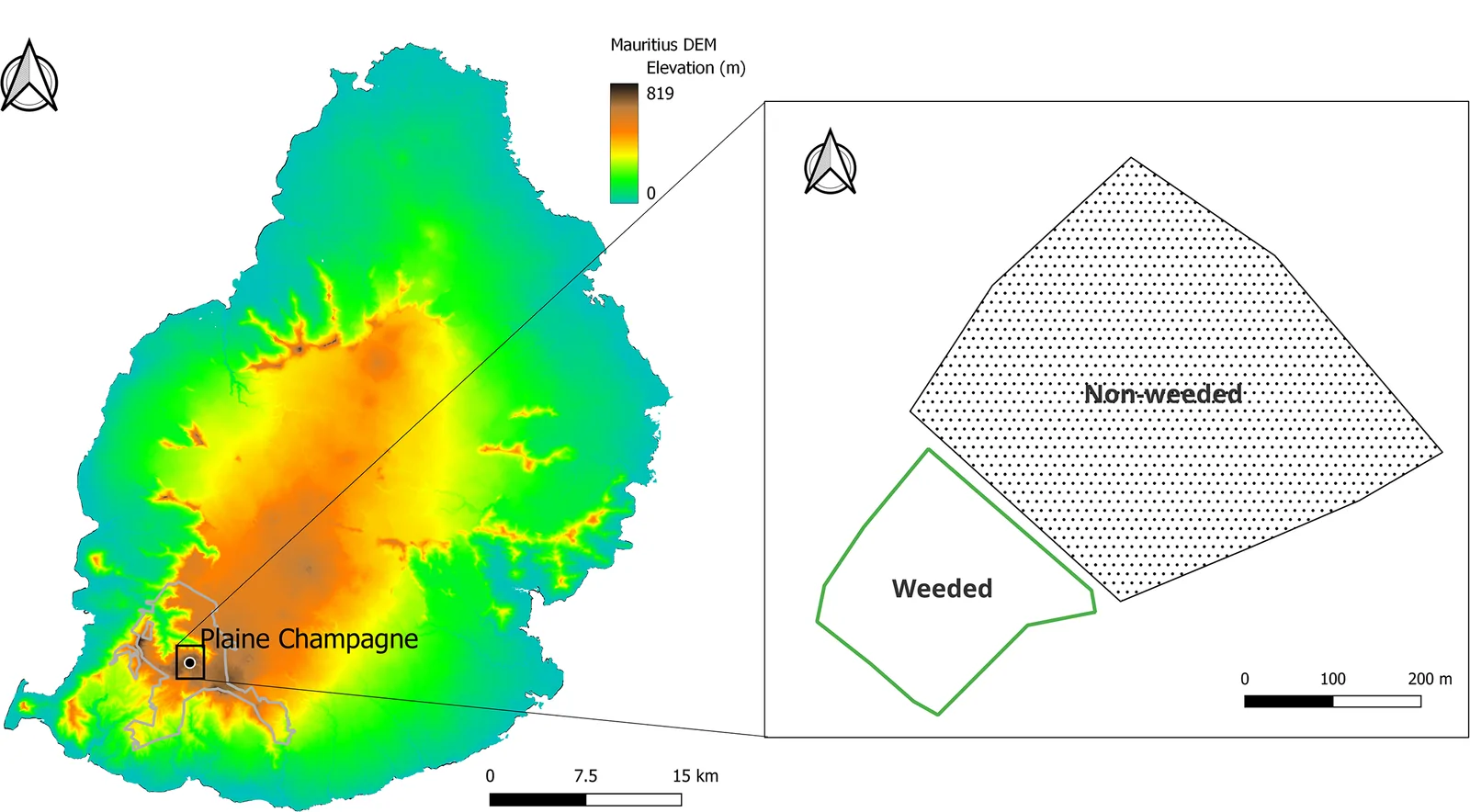
The study site of Plaine Champagne in Mauritius displayed on a digital elevation model (DEM) showing topographic variation (left). The Black River Gorges National Park is outlined. The outline of the weeded and invaded areas within Plaine Champagne sampled for this study is shown on the right.

We selected the four most abundant native tree species that occurred in both habitats, namely *Antirhea borbonica* J.F.Gmel. (Rubiaceae), *Labourdonnaisia calophylloides* Bojer (Sapotaceae), *Olea lancea* Lam. (Oleaceae), *Aphloia theiformis* (Vahl) Benn. (Aphloiaceae), along with *Psidium cattleyanum*, the dominant IAP in invaded areas, and *Harungana madagascariensis*, the dominant native pioneer tree species regenerating naturally following IAP removal. In each habitat, we sampled 15 individuals from each species between May and August 2025. For each individual tree, we assessed the incidence of three forms of folivory (chewing, mining, and gall making) on 30 selected leaves, and their severity on an additional 30 selected leaves (Materials S2 and S3). To do so, 60 leaves were collected haphazardly on each individual, deliberately avoiding oversampling from any single branch to ensure representativeness, before being pooled and subsequently separated at random for analysis. We defined herbivory incidence at plant level as the number of leaves affected divided by the total number of leaves sampled in a given plant. Herbivory severity was defined as the percentage of leaf area lost to each herbivory type combined. We used LeafByte v. 1.4.0 to measure leaf area and the percentage of chewing damage (Getman-Pickering et al., 2020). Mining and gall damage estimation was done by the same observer (first authors), with preliminary training on ZAX Herbivory Trainer (Xirocostas et al., 2022). After measuring total leaf area and the consumed leaf area, three leaves per plant were randomly selected from the same leaves that were used for herbivory measurements. These were dried at The Mauritius Herbarium and their dry weight (DW) measured. From these data, the specific leaf area (SLA) Index (in m^2^ kg^−1^) was calculated as the leaf area divided by the dry weight (Material S4).

Finally, the actual folivory rate (the percentage of leaf area damaged per month) was calculated (Material S5). Some species harbour leaves with a short lifespan, which can be shed before significant herbivory damage accumulates. In contrast, species with leaves having a long lifespan have more chances to accumulate herbivory damage before the leaves are shed. Hence, we monitored folivory over time for the above-mentioned selected species to evaluate whether accumulated folivory damage can serve as a good indicator of the folivory rate. For each species, 15 individuals ranging from 1 to 3.4 m were randomly selected. For each individual, 20 mature leaves were randomly tagged using a unique number written with permanent marker on the abaxial side of each leaf, and monitored monthly between July 2025 and January 2026. This monitoring period covers the dry winter and wet summer seasons. The folivory rate per plant was calculated as the average of the folivory rates of the sampled leaves of each plant. All leaves abscised before the last census were excluded for calculation. Folivory estimation was done by the same observers over the entire monitoring period and ZAX Herbivory Trainer (Xirocostas et al., 2022) was used to ensure the highest estimation accuracy.

### Data analysis

R version 4.4.1 (R Core Team, 2024) was used to do all statistical analysis and graphs. Graphs were produced using the R package ‘ggplot2’ (Wickham, 2016). Hill numbers (orders q = 0, 1, and 2) were calculated per trap and habitat type using the R package ‘HillR’ (Li, 2018) to quantify folivorous arthropod species richness (q = 0), the effective number of common species based on Shannon diversity (q = 1), and dominance based on Simpson diversity (q = 2) (Hill, 1973; Chao, Chiu & Jost, 2014). Differences in arthropod species richness and abundance among habitat types were assessed using generalized linear models, with habitat type included as the main explanatory variable. Count data were examined for overdispersion and zero inflation using simulation-based residual diagnostics implemented in the ‘DHARMa’ package (Hartig, 2024). Species richness was analyzed using Poisson models, while total arthropod abundance was modelled using negative binomial distributions. Model performance was evaluated using pseudo-R^2^ metric (1 − residual deviance/null deviance) (Zuur et al., 2009). To assess variation in habitat quality for arthropods, total species richness and total abundance per trap were used as response variables. All these analyses were done on data collected from 18 traps in invaded habitat and 20 in weeded habitat as two traps were lost. All graphical outputs were generated using the R package ‘ggplot2’ (Wickham, 2016).

To examine how herbivory is influenced by habitat types, we used general linear mixed-effect models (GLMMs). We used herbivory incidence and severity at plant level as response variables and first ran the models with habitat type as main predictor, and species and plant ID as random factors. For herbivory incidence, model diagnostics based on DHARMa simulations indicated neither zero inflation nor overdispersion (dispersion = 1.33), suggesting an adequate binomial model fit. Herbivory severity, expressed as proportional leaf area damaged, was analysed using beta regression with a logit link. Following this, the same models were run for each species individually, with habitat type as a fixed effect, and plant ID as a random factor to account for non-independence among leaves sampled on the same plant. Species occurring in only one habitat type (*P. cattleyanum* in the invaded area and *H. madagascariensis* in the area under restoration) were excluded from species-level analyses, as treatment effects could not be estimated in the absence of replication across habitats. In all cases, each full model including habitat type was compared to its respective null model containing only the random effect using likelihood ratio tests (LRTs) to assess the significance of habitat effects on herbivory incidence and severity. When a significant habitat effect was detected, estimated marginal means of herbivory incidence and severity were calculated for each habitat type using the emmeans package, and pairwise comparisons were performed on the log-odds scale with Tukey-adjusted *p*-values (Lenth, 2025).

The distribution of SLA, consumed area, and folivory rate data was assessed using the Shapiro–Wilk test. As the data deviated from normality, non-parametric tests were applied in subsequent analyses. SLA, consumed area, and folivory rate at plant level were compared between the different habitat type using Kruskal–Wallis rank sum tests. No *post-hoc* tests were performed, as only two treatments were present, and differences were interpreted from median values (Mangiafico, 2024; Dinno, 2024). For the purpose of these analyses, individuals of *H. madagascariensis* (dominant native pioneer species naturally regenerating after IAP control) and *P. cattleyanum* (dominant alien invasive species in invaded areas) were compared to evaluate their response in both habitats, while controlling for species-specific identity.

## Results

Overall, 3,306 arthropods from seven different Orders, including folivores, were recorded, including 1,545 individuals from seven Orders in the weeded forest and 1,761 individuals from seven Orders in the invaded forest. The most abundant Order was Hemiptera, accounting for 44.2% of all arthropods recorded. Hemiptera was also the most frequent Order recorded, occurring in 100% of samples, and Thysanoptera the least frequent, recorded in 31.6% of traps. At habitat level, Hemiptera was also the most abundant Order recorded, accounting for 35.3% of all arthropods recorded in the invaded area, and 54.3% in the weeded area. Diptera and Hemiptera were also the most frequently recorded Orders in the weeded forest, along with Coleoptera, Diptera, and Hymenoptera in the invaded forest, all of which occurred in all samples from the respective habitat type. Generally, invaded areas hosted invertebrate communities slightly more diverse, even, and showing a broader set of co-dominant species than weeded areas (Material S6). Nevertheless, habitat type (invaded *vs*. weeded) did not have a statistically significant influence on invertebrate species richness (Poisson GLM, likelihood ratio test: χ^2_1_^ = 0.34, *df* = 1, *p* = 0.558) or abundance (negative binomial GLM, likelihood ratio test: χ^2_1_^ = 1.04, *df* = 1, *p* = 0.307).

Table 1 provides the main results of the comparisons between invaded and weeded native forests. Habitat type significantly impacted herbivory incidence and herbivory severity at plant level. Plants in invaded areas exhibited higher odds of leaf damage (odds ratio = 1.57, *p* < 0.001) and higher herbivory severity (odds ratio = 1.36, *p* = 0.038) than those in weeded areas. At species level, habitat type significantly impacted herbivory incidence on *Antirhea borbonica*, and *Labourdonnaisia calophylloides*, but not on *Olea lancea* and *Aphloia theiformis* (Fig. 2). *Antirhea borbonica* exhibited higher odds of leaf damage in invaded compared to weeded areas (odds ratio = 11.1, *p* = 0.013), a trend also found in *L. calophylloides* (odds ratio = 2.37, *p* = 0.001). Habitat type also significantly impacted herbivory severity on *A. borbonica* and *A. theiformis*, but not on *L. calophylloides* (*p* > 0.05) and *O. lancea* (*p* > 0.05) (Fig. 3). *Antirhea borbonica* exhibited higher herbivory severity in invaded areas compared to weeded areas (odds ratio = 7.48, *p* = 0.001), but *A. theiformis* exhibited lower herbivory severity in invaded areas compared to weeded areas (odds ratio = 0.526, *p* = 0.006). Folivory rate and specific leaf area index (SLA) are similar for most species between habitat type. Folivory rate is however significantly higher on the dominant species in the weeded forest (*H. madagascariensis*) compared to the dominant species in the invaded forest (*P. cattleyanum*) (χ^2^ = 5.385, *df* = 1, *p* = 0.020) (Material S7). SLA, on the other hand, is significantly different between weeded and invaded areas only for *L. calophylloides* (χ^2^ = 21.793, *df* = 1, *p* < 0.001) and between *H. madagascariensis* and *P. cattleyanum* (χ^2^ = 53.041, *df* = 1, *p* < 0.001), with *L. calophylloides* having a significantly higher SLA in weeded areas and *H. madagascariensis* having a significantly higher SLA than *P. cattleyanum* (Fig. 4). Consumed leaf area differ between invaded and weeded areas only for *A. borbonica* (χ^2^ = 5.30, *df* = 1, *p* < 0.021), *A. theiformis* (χ^2^ = 8.79, *df* = 1, *p* = 0.003), and between *H. madagascariensis* and *P. cattleyanum* (χ^2^ = 21.77, *df* = 1, *p* < 0.001). *A. borbonica* had significantly larger areas of leaves consumed in weeded areas, and *A. theiformis* had less, while *H. madagascariensis* was significantly more consumed than *P. cattleyanum* (Fig. 5).

**Table 1:** Comparisons of herbivory incidence and severity between invaded and weeded native forests at habitat and species level in Plaine Champagne.

Response variable	Main predictor	Resolution	χ^2^	df	*p*-value
**Herbivory incidence**	Habitat type	Habitat	33.163	1	**<0.001**
		*Antirhea borbonica*	6.508	1	**0.011**
		*Aphloia theiformis*	0.950	1	0.330
		*Labourdonnaisia calophylloides*	9.171	1	**0.002**
		*Olea lancea*	1.860	1	0.173
**Herbivory severity**	Habitat type	Habitat	4.171	1	**0.041**
		*Antirhea borbonica*	14.198	1	**<0.001**
		*Aphloia theiformis*	6.968	1	**0.008**
		*Labourdonnaisia calophylloides*	0.052	1	0.820
		*Olea lancea*	0.457	1	0.499

**Note:**

Significant results are shown in bold text.

**Figure 2:**
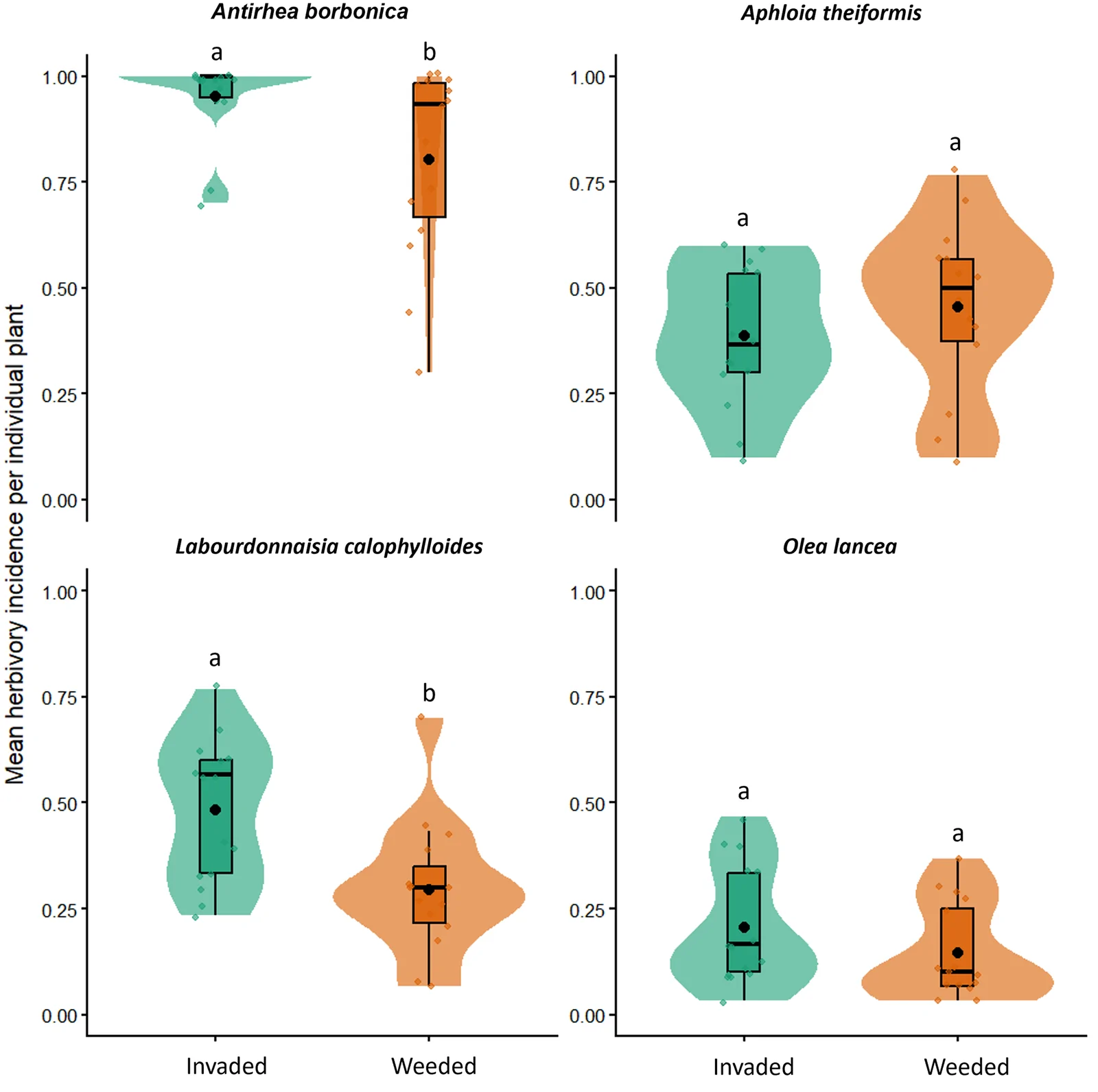
Mean invertebrate herbivory incidence (chewing, mining and galls) on selected native tree species in invaded and weeded forest areas in Plaine Champagne. Herbivory incidence is defined as the number of leaves affected divided by the total number of leaves sampled in a given plant. Letters above bars indicate statistical similarity or differences.

**Figure 3:**
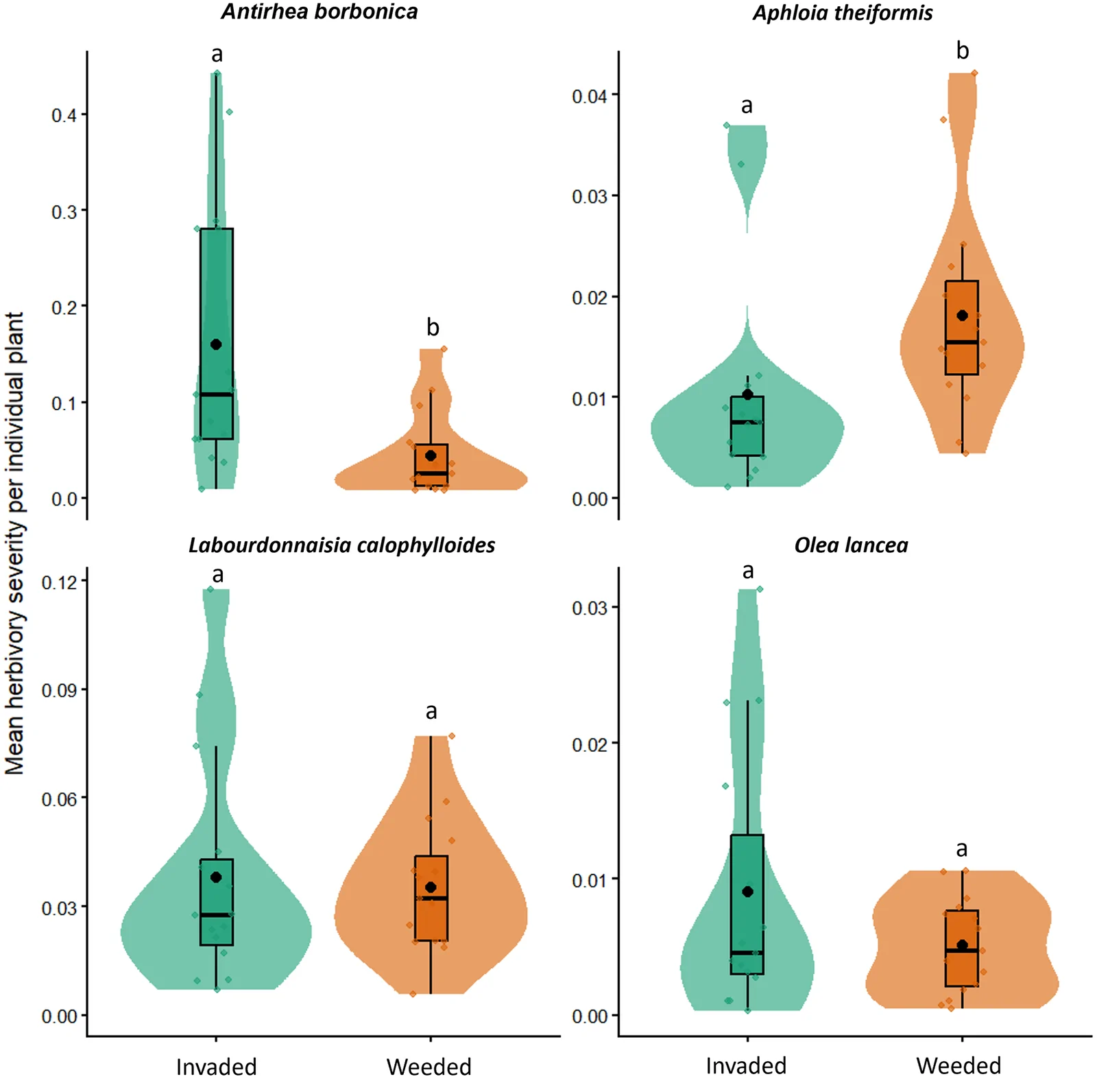
Mean invertebrate herbivory (chewing, mining and galls) severity on selected native tree species in invaded and weeded forest areas in Plaine Champagne. Herbivory severity is defined as the percentage of leaf area lost to each herbivory type combined. Letters above bars indicate statistical similarity or differences.

**Figure 4:**
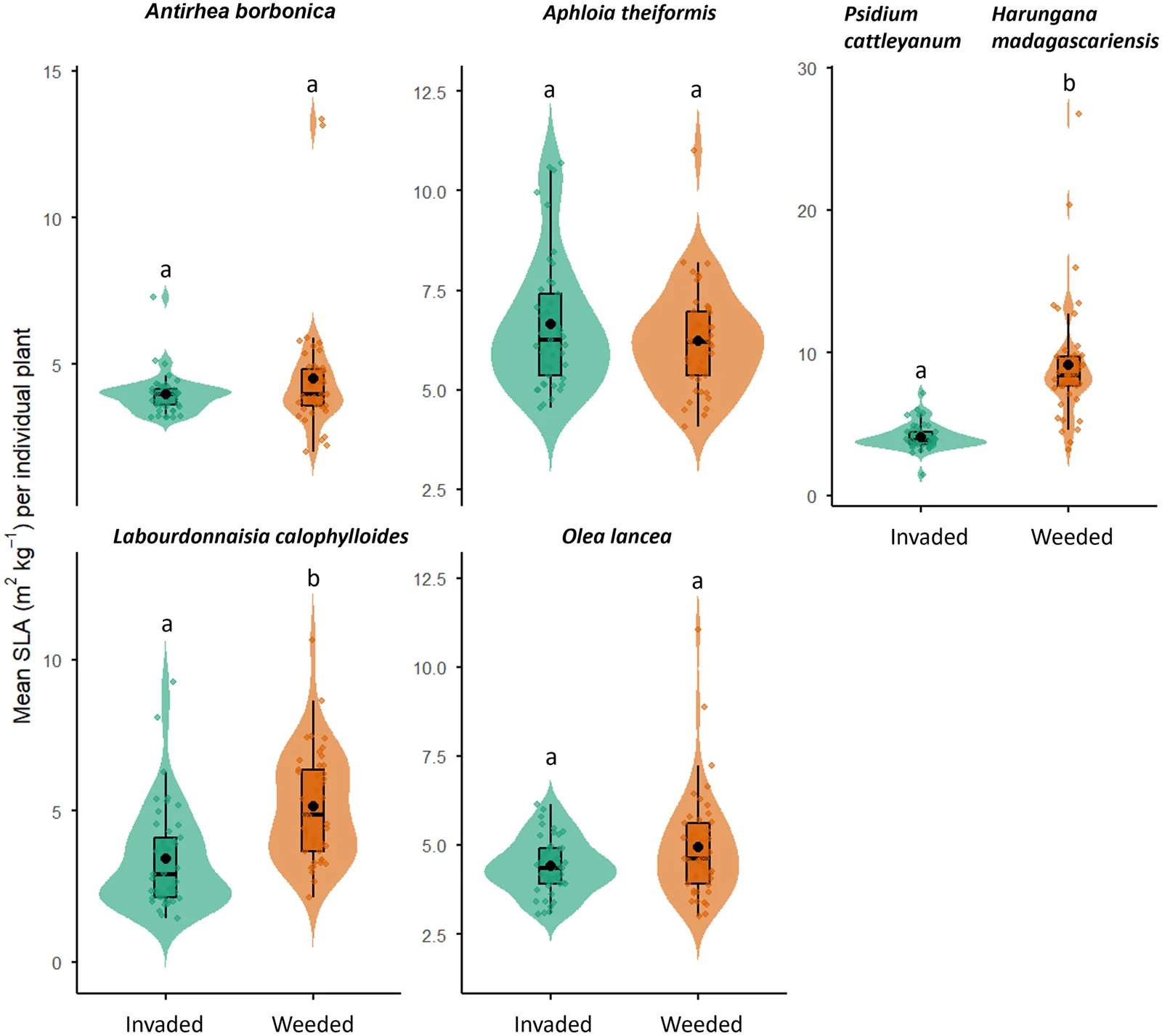
Specific Leaf Area (SLA) at plant level per species across habitat type in Plaine Champagne. *Psidium cattleyanum* represents the dominant invasive alien plant found in the invaded area and *Harungana madagascariensis* the dominant pioneer tree species found in the weeded area. Letters above bars indicate statistical similarity or differences.

**Figure 5:**
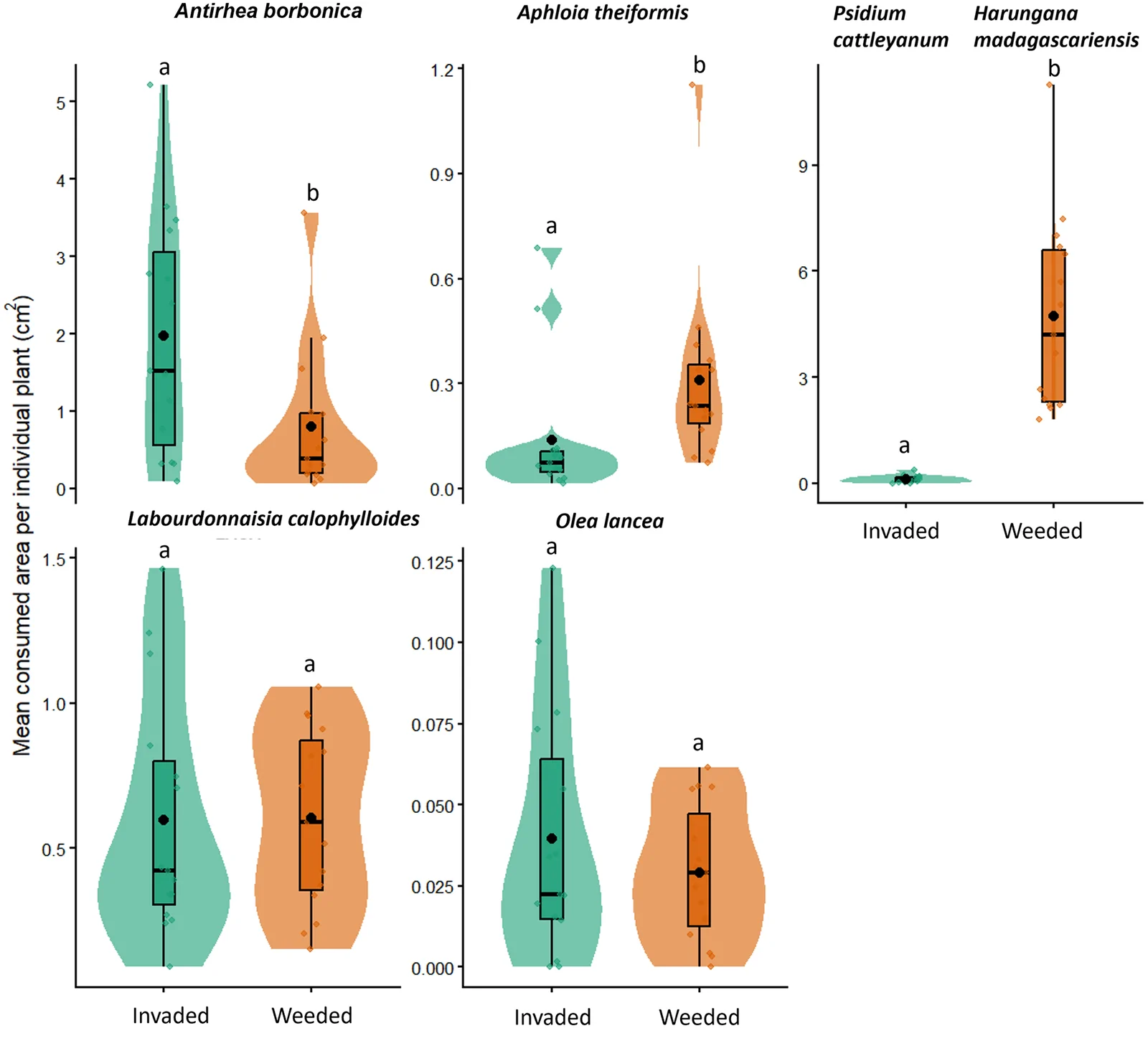
Mean consumed area of leaves by invertebrate folivores at plant level per species across habitat type in Plaine Champagne. *Psidium cattleyanum* represents the dominant invasive alien plant found in the invaded area and *Harungana madagascariensis* the dominant pioneer tree species found in the weeded area. Letters above bars indicate statistical similarity or differences.

## Discussion

### Ecological implications

Invasive alien plants impact arthropod communities in different ways, affecting the performance of native herbivores and their natural enemies such as parasitoids and predators (Bezemer, Harvey & Cronin, 2014; Smith & Schmitz, 2015), and their behaviour (Harvey, Bukovinszky & van der Putten, 2010; Ismail, Siemann & Ding, 2023). Such changes can influence the spatial and temporal dynamics of native arthropod populations and communities (Burghardt & Tallamy, 2013), ultimately leading to changes at the landscape level (Bezemer, Harvey & Cronin, 2014). Our results align with those studies as we reported structural differences in arthropod communities between invaded and weeded areas, although species richness and abundance did not differ significantly. Interestingly, the loss, through weeding, of a large amount of leaf tissue of alien plants, particularly of the highly dominant *P. cattleyanum* (Florens et al., 2016) did not lead to a significant drop in arthropod species richness and abundance. This suggests that the weeding of alien plants likely do not represent a significant resource loss for folivores, despite endemic folivores being known to eat leaves of IAP (Kitson et al., 2013), and in contrast with some other cases (Williamson & Fitter, 1996; Schlaepfer, Sax & Olden, 2011; Landsman, Schmit & Matthews, 2021). On the other hand, the invasive alien plants, are known to have detrimental effects on specific groups of invertebrate herbivores like butterflies (Florens et al., 2010), as well as on various other components of native biodiversity and functioning (Krivek et al., 2020; Bissessur et al., 2023).

Our results also show that IAP indirectly contribute to increased herbivory on native species, and can lead to greater structural investment in leaves in some cases (as seen for *L. calophylloides*), both of which might slow growth rates and potentially reduce fitness. Increased herbivory on native plants in invaded forests may arise through multiple, non-mutually exclusive mechanisms. First, invasion by IAP can reduce native plant diversity and simplify plant neighbourhoods (Baider & Florens, 2011; Florens et al., 2012, 2017; McCary et al., 2026), thereby weakening associational resistance, a process by which higher plant diversity reduces herbivore pressure on individual hosts (Barbosa et al., 2009; Stemmelen et al., 2022). Moreover, IAP often possess anti-herbivore physical and chemical defences to which native herbivores in the introduced range are not evolutionarily adapted (Cappuccino & Arnason, 2006; Ruiz-Guerra et al., 2021). For example, strawberry guava is known to develop thicker foliar cuticles, which likely reduces folivory, when growing in dense forest compared to more open forests (Medeiros, 2002). Furthermore, phenolics and terpenoids associated with allelopathy have also been identified in its leaves (Ho et al., 2012), and their impact on seed germination and root growth recorded (Hister, Trapp & Tedesco, 2016; Dacoreggio, Moroni & Kempka, 2019; Vasconcelos et al., 2019). These compounds have been widely recognised as multifunctional plant secondary metabolites that can reduce herbivory through antifeedant effects, impaired insect growth, digestive disruption, and oxidative damage to herbivore tissues (Mathesius, 2018; Singh, Kaur & Kariyat, 2021; Chatterjee et al., 2025).

Nevertheless, the lack of adaptation of native herbivore communities to such new plant defence mechanism could result in reduced herbivory on IAP (Keane, 2002; Liu & Stiling, 2006; Fu et al., 2024) and contribute to a corresponding spatial and functional shift of herbivore pressure towards native plant species (Zhai et al., 2024). Together, these processes suggest that IAP can indirectly increase herbivore pressure on native species that grow in the areas that they invade by reshaping both plant community composition and herbivore foraging behaviour. This new result is particularly important in the context of Mauritius, where native plant species already experience reduced fitness due to competition with IAP for resources (Baider & Florens, 2006; Bissessur et al., 2023; Monty, Florens & Baider, 2013), limiting their capacity to compensate for additional stress imposed by increased herbivory, and therefore risking further weakened performance that would indirectly facilitate the invasion success of IAP, especially of strawberry guava (Florens et al., 2016; Croy et al., 2020).

Finally, our results suggest that the removal of IAP improves folivore habitat quality by increasing the diversity and amounts of food resources. Indeed, this has been illustrated by a higher Specific Leaf Area for *L. calophylloides* in the weeded site, but also for *H. madagascariensis* compared with *P. cattleyanum*. These results indicate a higher provision of more palatable leaves following weeding of IAP, especially from the abundant natural regeneration of native pioneer species post-weeding. The removal of IAP therefore provides benefits both at the species and habitat level, starting in the early stages of restoration. We highlight locally hitherto undocumented mechanisms whereby IAP removal in ecological restoration projects benefit native biodiversity. With some rare exceptions like the — at least temporary — drawbacks documented on some molluscs groups (Florens, Daby & Jones, 1998) our results align well with previous studies showing similar ultimate positive outcomes of alien plant control for native biodiversity (Baider & Florens, 2011; Baguette, Baider & Florens, 2025b; Bissessur et al., 2023; Seeburrun, Baider & Florens, 2026). This is especially important in the context of small oceanic islands whose biodiversity often face extreme pressure (Caujapé-Castells et al., 2010; Hammond et al., 2015), and where studies on how invasive alien species influence ecological interactions are particularly rare (Seeburrun et al., 2026).

### Management implications

Our results indicate that IAP control has wider positive impacts on native plants and arthropods than previously known. This is particularly important because IAP are inexorably progressing in Mauritius (Florens et al., 2016), driving native biodiversity loss (Florens et al., 2017) over an area of native habitats that is over ten-folds that over which IAP have been controlled and are recovering (Republic of Mauritius, 2024). Consequently, there exists a net loss of native biodiversity *in-situ*. IAP control therefore deserves greater importance particularly because an increasing number of species are or are becoming threatened with extinction (Motala et al., 2007; BGCI, 2021; Cottrill et al., 2024; BirdLife International, 2026) or being declared Extinct (Tatayah et al., 2025), and habitats continue to degrade (Florens, 2013; Koenig & Deenapanray, 2024), even within protected areas (Florens et al., 2017; Bissessur et al., 2023). If the current loss of biodiversity is to be stopped, a paradigm shift is required to make conservation practitioners devote a substantially larger component of their efforts to controlling IAP over much larger areas than the current ~700 ha (8.5% of remnant native habitats) (Republic of Mauritius, 2024). The current study should support that shift as it uncovered new mechanisms hitherto unrecorded in Mauritius, by which alien plants are detrimental to native biodiversity, in addition to already recorded impacts (Baider & Florens, 2011, 2022).

In addition, our results provide further evidence of the detrimental impacts of cutting down native pioneer trees from areas undergoing restoration for conservation than had previously been recorded (Florens & Baider, 2013; Baguette, Baider & Florens, 2025a, 2025b). Native pioneer trees are known to be particularly beneficial to herbivore communities because of their ability to produce large quantities of palatable leaves and their typically limited defenses against herbivores (Coley, Bryant & Chapin, 1985; Poorter et al., 2004; Peñuelas et al., 2013; Mariano, Martínez-Garza & Alcalá, 2018). *Harungana madagascariensis* is a typical pioneer species that grows back naturally from the seed bank in severely degraded wet native forests after weeding is done for ecological restoration in Mauritius. Yet, most major conservation practitioners locally frequently cut down or recommend cutting the species from conservation management areas (Baguette, Baider & Florens, 2025b) on the basis of suppositions that the species is harmful to native biodiversity (Baguette et al., 2026). The premises of the suppositions have not only contradicted best available knowledge and ecological theory from the onset, but are also now increasingly contradicted by mounting scientific evidence to the contrary (Baguette et al., 2026). This study adds further support that cutting *H. madagascariensis* is detrimental to folivores in native forests. Shifting away from the currently highly interventionist, species-centred and supposition-based approach (Florens, 2013; Seeburrun et al., 2026) would also benefit native biodiversity better (Baguette, Baider & Florens, 2025a, 2025b).

Native forest restoration in Mauritius comprises a large share of planting seedlings within weeded forests where native pioneer species are often cut down, which is often followed by limited monitoring (Baguette et al., 2026) making the efficacy of the approach largely impossible to assess objectively relative to the extra cost it entails. We now show that the native pioneer trees that are often cut down, in fact provide abundant resources to forest folivores. We recommend that conservation practitioners stop the policy of cutting native pioneer trees, in order not to degrade habitat quality for herbivores. This would strengthen plant herbivore networks which would reasonably contribute towards preserving endemic species relying on invertebrates as food source, such as insectivorous bats (Bunsy, Vincenot & Florens, 2026), birds (Wijnhorst et al., 2024) and reptiles (Buckland et al., 2014). Expanding weeded areas while enabling or fostering natural regeneration of native pioneer trees should reduce reinvasion rates (Fehmi, Rasmussen & Arnold, 2021; Byun, 2023) while strengthening herbivore-plant interactions (Alavez et al., 2023), which in turn would benefit numerous organisms that depend on them. This would shift conservation policy and management closer to what truly benefits native biodiversity *sensu lato* instead of the current dominating view among conservation practitioners of overwhelmingly regarding native forests mostly as a collection of woody plants with little regards to other species like epiphytes (Baguette, Baider & Florens, 2025b) or herbivorous invertebrates (this study).

### Study strength and limitations

Mauritius was selected as a model island system because, following its relatively recent human colonization and extensive historical degradation, it pioneered ecological restoration through invasive alien species control from the 1930s (Vaughan & Wiehe, 1941; Cheke & Hume, 2008). Due to the historical extent of habitat loss and degradation, and the scarcity of sites where natural regeneration of native pioneer species like *Harungana madagascariensis* is not suppressed by conservation practitioners, this study could only be carried out at one site within adjacent habitat blocks. Limitations inherent to such a design were minimised by ensuring that the native structure and composition of the vegetation in areas being compared were similar, minimising the likelihood that observed patterns could be confounded by site-specific historical, geographical, and biological differences. Similarly, we restricted arthropods identification to Order level as there was no in-country insect specialist. Based on Biaggini et al. (2007) and Driessen & Kirkpatrick (2019), this approach allowed us to provide a broad indication of arthropod assemblage composition, and a useful first approximation of community-level differences between invaded and weeded habitats. Future studies could focus on precisely identifying the folivores responsible for leaf damage on focal plant species. Notwithstanding these constraints, our results provide strong evidence of ecological association between IAP and folivory dynamics, offering novel insights into the role of native pioneer trees (here *H. madagascariensis*), as an additional trophic resource following IAP removal. These corroborate an increasing body of scientific literature supporting its ecological status as a pioneer species and its numerous benefits for biodiversity conservation and ecological restoration (Baguette, Baider & Florens, 2025a, 2025b).

Overall, the major strength of this study lies in the depth and complementarity of its approach: by integrating arthropod community data with assessments of folivory impacts from multiple yet convergent perspectives, namely the incidence and severity of folivory on native species, and resource availability as a structuring force. This cross-sectoral approach, which remains uncommon in the ecological restoration literature (Ebeling et al., 2014), yields additional insight into the broader impacts of IAP on native species, ecosystems and key ecological interactions such as folivory, and their recovery potential once IAP are controlled. Collectively, our results provide actionable insights into the ecological importance of IAP control in island restoration contexts, and underscore the value of retaining and promoting pioneer species as facilitators of ecological interaction reinstatement, rather than treating them as targets for removal.

## Conclusion

We combined arthropod sampling with estimates of folivory to compare native forests that are invaded and weeded of IAP on the oceanic island of Mauritius. We found that IAP control represents negligible loss for arthropods and elicits several advantages for ecological restoration aimed at biodiversity conservation. We provide empirical evidence that weeding of IAP decrease folivory damage on native species, likely improving their fitness. The control of IAP also fosters the natural regeneration of native pioneer trees, which substantially increases resources for folivores, therefore improving their habitat quality. These results have several implications for conservation management. The most important one is that habitat restoration through IAP control should be given greater consideration in ecological restoration programmes in Mauritius, as the vast majority of its native plant species are threatened and declining. Second, it reinforces the judiciousness of using evidence-based approaches to inform ecological restoration, and underscores the detrimental effects of supposition-based conservation management, like controlling native pioneer tree species like *H. madagascariensis*. In conclusion, capitalising on IAP control and avoiding to cut down native pioneer tree species would be even more beneficial than previously reported, and help free already scarce conservation resources that may then be attributed to control IAP over larger areas of native forests.

## Supplemental Information

10.7717/peerj.21908/supp-1 Supplemental Information 1 Arthropod survey abundance matrix used for data analysis.

10.7717/peerj.21908/supp-2 Supplemental Information 2 Herbivory incidence raw data.

10.7717/peerj.21908/supp-3 Supplemental Information 3 Herbivory severity and consumed area raw data.

10.7717/peerj.21908/supp-4 Supplemental Information 4 Leaf Mass Area raw data used for analysis.

10.7717/peerj.21908/supp-5 Supplemental Information 5 Folivory rate data.

10.7717/peerj.21908/supp-6 Supplemental Information 6 Effective number of species (Hill numbers) per trap across habitat types in Plaine Champagne (weeded and invaded). q0 represents the mean species richness, q1 the mean effective number of equally common species, and q2 the mean effective number of dominant species. The blue line represents the invaded area while the orange line represents the weeded area.

10.7717/peerj.21908/supp-7 Supplemental Information 7 Violin plots showing the mean folivory rate (the percentage of leaf area damaged per month) at plant level per species across habitat type (Invaded *VS* Weeded) in Plaine Champagne. ‘ANBO’ refers to *A. borbonica*, ‘APTH’ to *A. theioformis*, ‘LACA’ to *Labourdonnaisia calophylloides*, ‘OLLA’ *Olea lancea* and ‘DOM’ to *Psidium cattleyanum* within the invaded area and *Harungana madagascariensis* in the weeded area. Letters above bars indicate statistical similarity or differences.

10.7717/peerj.21908/supp-8 Supplemental Information 8 Codebook.

## Additional Information and Declarations

### Field Study Permissions

The following information was supplied relating to field study approvals (*i.e*., approving body and any reference numbers):

Only specimens of arthropods were collected at the study site for identification purposes. As this group of organisms does not fall within the regulated animals, ethical approval was not required.

### Data Availability

The following information was supplied regarding data availability:

The data is available in the Supplemental Files.
